# Development and characterization of narsoplimab, a selective MASP-2 inhibitor, for the treatment of lectin-pathway–mediated disorders

**DOI:** 10.3389/fimmu.2023.1297352

**Published:** 2023-11-08

**Authors:** Thomas Dudler, Sadam Yaseen, W. Jason Cummings

**Affiliations:** Discovery, Omeros Corporation, Seattle, WA, United States

**Keywords:** narsoplimab, MASP-2, lectin pathway, complement, pharmacokinetics, pharmacodynamics

## Abstract

**Introduction:**

Overactivation of the lectin pathway of complement plays a pathogenic role in a broad range of immune-mediated and inflammatory disorders; mannan-binding lectin-associated serine protease-2 (MASP-2) is the key effector enzyme of the lectin pathway. We developed a fully human monoclonal antibody, narsoplimab, to bind to MASP-2 and specifically inhibit lectin pathway activation. Herein, we describe the preclinical characterization of narsoplimab that supports its evaluation in clinical trials.

**Methods and results:**

ELISA binding studies demonstrated that narsoplimab interacted with both zymogen and enzymatically active forms of human MASP-2 with high affinity (K_D_ 0.062 and 0.089 nM, respectively) and a selectivity ratio of >5,000-fold relative to closely related serine proteases C1r, C1s, MASP-1, and MASP-3. Interaction studies using surface plasmon resonance and ELISA demonstrated approximately 100-fold greater binding affinity for intact narsoplimab compared to a monovalent antigen binding fragment, suggesting an important contribution of functional bivalency to high-affinity binding. In functional assays conducted in dilute serum under pathway-specific assay conditions, narsoplimab selectively inhibited lectin pathway-dependent activation of C5b-9 with high potency (IC_50_ ~ 1 nM) but had no observable effect on classical pathway or alternative pathway activity at concentrations up to 500 nM. In functional assays conducted in 90% serum, narsoplimab inhibited lectin pathway activation in human serum with high potency (IC_50_ ~ 3.4 nM) whereas its potency in cynomolgus monkey serum was approximately 10-fold lower (IC_50_ ~ 33 nM). Following single dose intravenous administration to cynomolgus monkeys, narsoplimab exposure increased in an approximately dose-proportional manner. Clear dose-dependent pharmacodynamic responses were observed at doses >1.5 mg/kg, as evidenced by a reduction in lectin pathway activity assessed *ex vivo* that increased in magnitude and duration with increasing dose. Analysis of pharmacokinetic and pharmacodynamic data revealed a well-defined concentration-effect relationship with an *ex vivo* EC_50_ value of approximately 6.1 μg/mL, which was comparable to the *in vitro* functional potency (IC_50_ 33 nM; ~ 5 μg/mL).

**Discussion:**

Based on these results, narsoplimab has been evaluated in clinical trials for the treatment of conditions associated with inappropriate lectin pathway activation, such as hematopoietic stem cell transplantation-associated thrombotic microangiopathy.

## Highlights

We developed narsoplimab, a fully human monoclonal antibody against MASP-2, to treat conditions associated with pathogenic activation of the lectin pathway of complement. Herein, we describe the characterization of narsoplimab in preclinical studies that supports its evaluation in clinical trials.

## 1 Introduction

**Figure 1:**
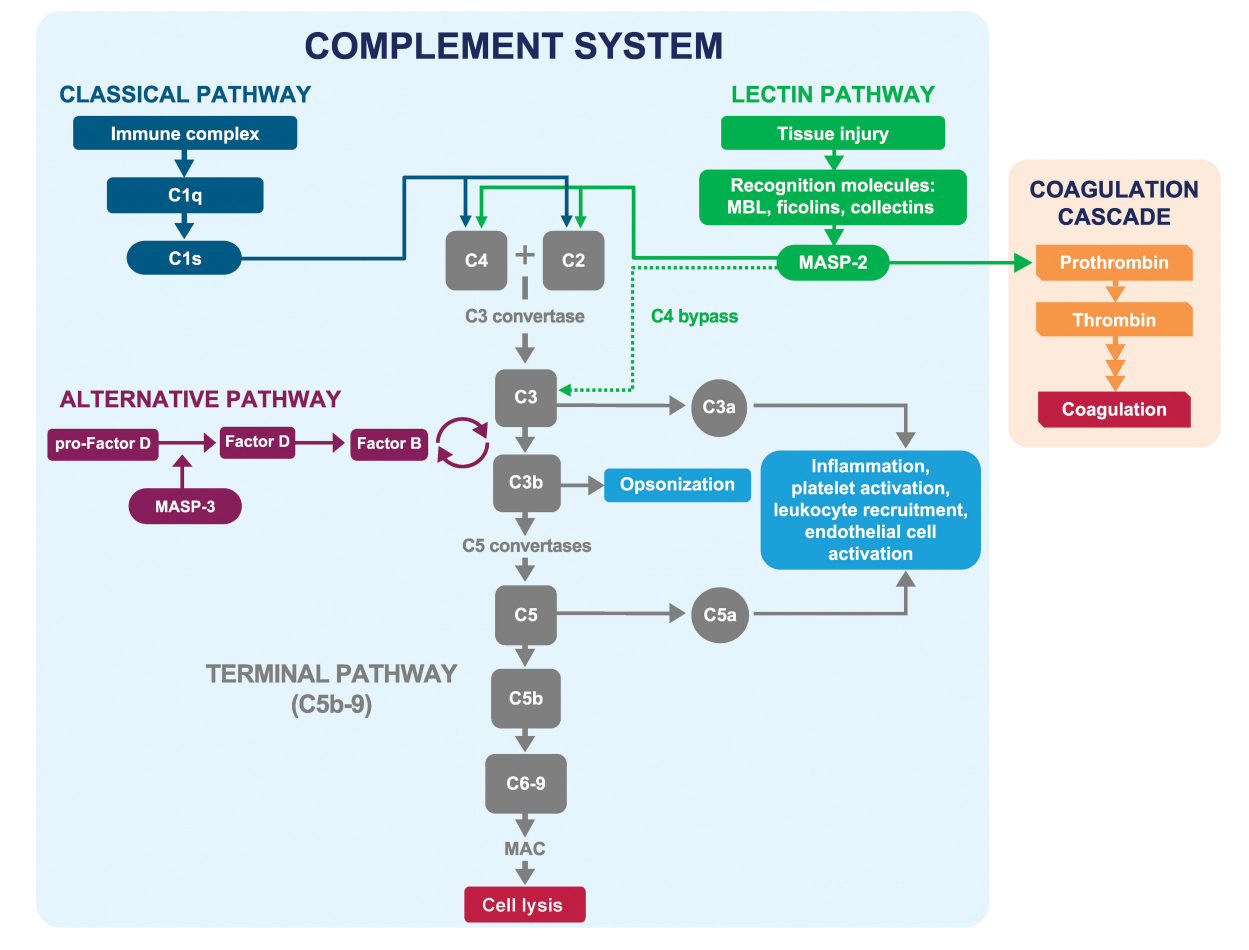
Classical, lectin, and alternative pathways of the complement system. The classical, lectin, and alternative pathways of complement converge at C3, which is cleaved to activate C5 and initiate formation of the membrane attack complex (C5b-9). The lectin pathway is activated in response to patterns present on pathogens and injured host cells. MASP-2 is the key effector enzyme of the lectin pathway and is activated following surface sequestration via associated PRMs (MBL, ficolins-1, 2, and 3, collectin-10, and collectin-11) binding to carbohydrate and aberrant glycosylation patterns on pathogens and injured host cells. Activated MASP-2 cleaves C4 and C2, resulting in the formation of the C3 convertase C4b2b, which activates C3. MASP-2 also activates the coagulation cascade through cleavage of prothrombin to thrombin and activation of Factor XII. Adapted from Gavriilaki E et al. Exp Hematol Oncol 2021; 10:57. MAC, membrane attack complex (C5b-9); MASP, mannan-binding lectin-associated serine protease; MBL, mannan-binding lectin; PRM, pattern-recognition molecule.

The complement system is a key component of innate immunity. However, overactivation of complement can play a pathogenic role in many diseases, including hemolytic disorders, thrombotic microangiopathies (TMAs), renal diseases, and a broad range of other complement-mediated inflammatory diseases or “complementopathies” (1–4). Three distinct pathways activate the complement system: the classical pathway, the lectin pathway, and the alternative pathway (**Figure 1**) (5, 6). The classical pathway, which is triggered mainly by immune complexes, serves as a “bridge” between innate and adaptive immunity (7). The lectin pathway is triggered by molecular patterns that are present on pathogens or damaged host tissue and bind to pattern-recognition molecules, initiating the lectin pathway (8–10). The alternative pathway is primarily an amplification loop for the other pathways; excessive complement activation occurs via dysregulation of this pathway (11). All three pathways converge on complement component C3, which is cleaved to further activate C5 and the terminal complement pathway, resulting in formation of the membrane attack complex (C5b-9).

Substantial efforts have been devoted to developing therapeutics targeting individual complement factors or activation pathways, including C5 inhibitors (e.g., eculizumab, ravulizumab) (12, 13), C3 inhibitors (e.g., pegcetacoplan) (14), alternative pathway inhibitors (e.g., iptacopan, vemircopan) (15, 16), and classical pathway inhibitors (e.g., sutimlimab) (17). Inhibiting the classical pathway compromises immunity because this pathway provides important immune effector function for antibodies directed against infectious agents (7). Blocking C3 or C5 also reduces serum bacteriolytic activity with or without vaccination (18) and may increase the risk of infection. For example, the C5 inhibitors eculizumab and ravulizumab have boxed warnings for serious meningococcal infections, and these therapies are available in the United States only through a restricted program under a Risk Evaluation and Mitigation Strategy (19, 20).

Selectively targeting the lectin pathway has the potential to reduce complement activation in damaged tissues without disrupting the beneficial role of complement in fighting infection. The key effector enzyme of the lectin pathway is mannan-binding lectin-associated serine protease-2 (MASP-2) (21), a serine protease exclusively produced by the liver and excreted into the circulation as a catalytically inactive zymogen (22–24). In plasma, MASP-2 is found predominantly in association with pattern recognition molecules (PRMs) collectively termed lectins, including mannan-binding lectin (MBL), ficolins-1, 2, and 3, collectin-10, and collectin-11 (25). These PRMs bind to non-self molecular patterns, such as glycosylation patterns or acetylated moieties present on pathogens and injured host cells but not present on healthy host tissue (6, 26–31). Binding of these PRMs to their cognate ligands leads to the juxtaposition of PRM-MASP complexes on activating surfaces, resulting in proteolytic conversion of MASP-2 zymogens into their enzymatically active form (32–34). While MASP-2 can auto-activate in MASP-1–deficient serum (21), under normal conditions, the MASP-2 cleavage reaction is catalyzed predominantly by MASP-1 (34). Activated MASP-2 cleaves C4 and C2, resulting in the formation of the C3 convertase C4b2b (formerly “C4b2a”). This initiates a series of enzymatic steps that results in activation of C3 and C5, leading to release of anaphylatoxins C3a and C5a, deposition of C3b, and formation of the membrane attack complex C5b-9 (**Figure 1**) (33, 35). MASP-2–dependent complement activation through C3 activation may also occur independently of C4 and C2 (36).

Experimental studies in mice have demonstrated that targeting MASP-2 allows for selective inhibition of the lectin pathway of complement, leaving the classical and alternative pathways intact and fully functional (21). Additional studies have demonstrated that deficiency or blockade of MASP-2 improves outcomes in experimental models of myocardial infarction (37), stroke (38), transplantation (39), kidney fibrosis (40), traumatic brain injury (41), and age-related macular degeneration (42, 43). Selective inhibition of the lectin pathway using MASP-2 inhibitors could potentially treat various lectin-mediated disorders while minimally impairing the protective role of complement in fighting infections (44, 45).

We developed narsoplimab (OMS721), a fully human immunoglobulin gamma 4 (IgG4) monoclonal antibody, to bind to MASP-2 and specifically inhibit lectin pathway activation. In this report, we summarize a series of preclinical studies to describe the pharmacologic activity of narsoplimab. We further describe the functional characterization of narsoplimab under near-physiologic conditions in multiple species and *ex vivo* pharmacodynamic response assessments in primates treated with narsoplimab. These studies provide the basis for clinical studies of narsoplimab.

## 2 Materials and methods

Narsoplimab was derived from a precursor scFv antibody isolated from a naïve human phage display library followed by light-chain shuffling and conversion to a full-length human immunoglobulin of IgG4 isotype (46). The narsoplimab test material used in this study was purified from supernatants of a Chinese hamster ovary (CHO) cell line stably transfected with expression constructs encoding the heavy and light chains of narsoplimab. Cells were grown in PF-CHO media for 16 to 20 days and cell-free supernatant was collected when cell viability dropped below 50%. Narsoplimab was purified by protein A affinity chromatography followed by ion exchange, concentration, and buffer exchange into phosphate-buffered saline (PBS). For monovalent interaction studies, a monovalent antigen-binding fragment (Fab) was isolated from a functionally equivalent IgG2 isotype variant of narsoplimab using the Pierce™ Fab preparation kit (Thermo Scientific) following the manufacturer’s recommendations. The IgG2 isotype variant was employed due to resistance of narsoplimab to the papain cleavage necessary for Fab preparation. Full-length recombinant human MASP-2 comprises an active site serine-to-alanine mutation and a C-terminal His tag was used as antigen. The material was isolated by nickel-affinity chromatography from stably transfected CHO cell supernatants and suspended at 0.253 mg/mL in phosphate buffered saline at pH 7.4. A catalytically active MASP-2 fragment comprising the CCP1-CCP2-SP domains was prepared as described elsewhere (47).

### 2.1 Characterization of the interaction of narsoplimab with MASP-2

A series of surface plasmon resonance studies was conducted to characterize the monovalent and bivalent interaction of narsoplimab with human MASP-2 using a BiaCore 3000 (GE Healthcare Life Sciences) instrument. MASP-2 antigen was immobilized on the glass surface by direct amine coupling, and the association and dissociation kinetics of intact narsoplimab or monovalent Fab were assessed at 25°C using 10 mM HEPES, 150 mM NaCl, 0.01% Tween-20, pH 7.4 as the mobile phase. Experimental data were globally fitted to a 1:1 interaction model to estimate association (k_a_) and dissociation (k_d_) rate constants, which were subsequently used to calculate the equilibrium dissociation constant (*K_D_*) for the narsoplimab–MASP-2 interaction (*K_D_* = k_d_/k_a_).

To obtain *K_D_* values for the binding of intact narsoplimab or monovalent Fab to MASP-2 by ELISA, polystyrene plates were coated overnight at 2°C to 8°C with recombinant human MASP-2 as antigen. Any remaining sites were blocked with bovine serum albumin, then serially diluted narsoplimab or Fab was added to the antigen-coated wells and incubated for 1 hour. After washing the wells to remove unbound material, a horseradish peroxidase-conjugated anti-human IgG detection antibody was added to all wells. Incubation was followed by washing to remove unbound conjugate, then 3,3′,5,5′-tetramethylbenzidine (TMB) substrate was added to the wells for color development. The development step was stopped with acid and the extent of TMB substrate conversion was quantitated colorimetrically by measuring the absorbance at 450 nm. The *K_D_* was estimated by fitting the binding dose-response data to a 4-parameter logistic model using GraphPad Prism version 6.02.

### 2.2 Specificity and selectivity of narsoplimab

The specificity of narsoplimab for human MASP-2 relative to four other serine proteases of the complement system (C1r, C1s, MASP-1, and MASP-3) in human serum was assessed by solid-phase ELISA following the procedure described above. Briefly, the serine proteases were immobilized on polystyrene plates, then allowed to react with different concentrations of narsoplimab. Bound antibody was revealed by peroxidase-labelled detection antibody and quantified by absorbance measurements. Apparent *K_D_* values were estimated by nonlinear regression using a four-parameter logistic model.

To assess selectivity, the effects of narsoplimab on lectin, classical, and alternative pathway-dependent activation of C5b-9 were compared using the Wieslab^®^ Complement System Screen ELISA kit (Svar Life Science, Malmö, Sweden). Human serum samples, diluted into pathway-specific assay buffers following the manufacturer’s instructions, were preincubated with different concentrations of narsoplimab followed by exposure of serum antibody mixtures to the appropriate pathway-specific activation wells provided by the kit. C5b-9 activation was evaluated using alkaline phosphatase-labeled antibody specific for the neoantigen exposed upon C5b-9 formation.

### 2.3 Pharmacologic activity of narsoplimab *in vitro*

Traditional methods to evaluate lectin pathway activity use highly diluted serum samples (typically 1% serum). While simple and robust, the concentrations of all components controlling the complement activation response in assays using highly diluted serum are approximately 100-fold lower than their respective *in vivo* concentrations, and evaluations of MASP-2 inhibitors such as narsoplimab in these assay conditions may not appropriately characterize their pharmacologic activity under physiologic conditions. To assess pharmacologic activity under near-physiologic conditions *in vitro*, ELISA and flow cytometry-based test methods using minimally diluted serum samples from mice, rabbits, cynomolgus monkeys, and rats as test matrix were developed.

In separate 96-well microtiter plates, minimally diluted (90%) serum samples from mice, rabbits, cynomolgus monkeys, and rats were pre-incubated with serially diluted narsoplimab (up to 500 nM). For ELISA assays, lectin-induced complement deposition reactions were initiated by addition of serum antibody preincubation mixtures to the mannan-coated assay plates. For flow cytometry assays, deposition reactions were initiated by the addition of 2.5 μL of mannan-coated beads (~250,000 beads) to antibody-serum preincubation mixtures. Following incubation at the indicated reaction time and temperature, reactions were stopped and developed for C3 or C4 activation as described below. During method development, time-course studies were conducted to analyze reaction kinetics of the lectin pathway activation response for the test matrix from each species, and a reaction time during the linear phase of the reaction that provided a robust complement activation signal in the absence of narsoplimab was selected for pharmacologic assessments. Lectin pathway activation was very rapid and proceeded to completion within minutes when evaluated at 37°C (data not shown). Therefore, assays were conducted at 4°C to achieve reaction kinetics compatible with standard benchtop plate-based assay procedures.

At the end of the incubation period, the reactions were stopped with ice-cold buffer and the plates (ELISA) or beads (flow) were washed twice. After the wash steps, lectin-induced complement activation was quantified using a primary antibody suitable for the relevant test species. For C3 activation, the wells or beads were incubated for 1 hour at room temperature with rabbit anti-human C3c antibody (which recognizes C3b and C3c) for all species. For C4 activation by ELISA, the wells were incubated for 1 hour at room temperature with biotinylated anti-mouse C4 antibody for mouse serum and with biotinylated chicken anti-human C4 antibody for monkey, rabbit, and human serum. For C4 activation by flow cytometry, the beads were incubated for 1 hour at 4°C with biotinylated anti-mouse C4 antibody for mice, with biotinylated chicken anti-human C4 antibody for rabbits, and with mouse anti-human C4d monoclonal antibody (which recognizes C4b as well as its degradation product C4d) for humans and monkeys.

The functional potency of MASP-2 inhibition (pharmacologic activity) was estimated by determining the half-maximal inhibitory concentration (IC_50_) for the narsoplimab inhibition dose-response curve for activation of C3 or C4. IC_50_ values were estimated by plotting the absorbance values (ELISA) or mean fluorescent intensity values (flow cytometry) against the antibody concentration and then fitting the data to a 4-parameter logistic regression.

### 2.4 Pharmacokinetic/pharmacodynamic relationship of narsoplimab in cynomolgus monkeys

To characterize the pharmacokinetics and pharmacodynamics of narsoplimab in primates, cynomolgus monkeys were treated with a single narsoplimab dose of 0.05, 0.15, 0.5, 1.5, or 5 mg/kg administered by intravenous (IV) bolus injection. Serial serum samples were collected predose and approximately 1, 24, 96, 168, 336, and 672 hours after narsoplimab administration. Additional serum samples were collected at 0.083, 0.25, 3, 6, 48, 240, and 504 hours after narsoplimab administration for monkeys exposed to 5 mg/kg. Narsoplimab serum concentrations were measured using a human IgG4 sandwich ELISA kit (Cayman Chemical, Ann Arbor MI) following the manufacturer’s instructions.

The pharmacodynamic response to narsoplimab treatment was evaluated by quantifying changes in *ex vivo* lectin pathway activity in response to narsoplimab treatment using an ELISA method similar to that employed for *in vitro* pharmacologic activity assessment (see Section 2.3). To calculate the pharmacodynamic response (% lectin pathway inhibition), the residual *ex vivo* lectin pathway activity (C4 activation by mannan) in serum samples collected after narsoplimab administration was quantified and expressed relative to the *ex vivo* lectin pathway activity in serum samples from the same animal at baseline using the following formula: % lectin pathway inhibition = 100 * (lectin pathway activity [baseline] – lectin pathway activity [postdose])/lectin pathway activity (baseline).

To characterize the pharmacokinetic/pharmacodynamic relationship in cynomolgus monkey, the complete set of drug concentration and pharmacodynamic response (% lectin pathway inhibition) data were fitted using a 4-parameter logistic regression model to estimate the EC_50_ value.

### 2.5 Animal welfare

Animal welfare complied with the U.S. Department of Agriculture’s (USDA) Animal Welfare Act (9 CFR Parts 1, 2 and 3). The Guide for the Care and Use of Laboratory Animals, Institute of Laboratory Animal Resources, National Academy Press, Washington, D.C., was followed. The facilities maintained an Animal Welfare Assurance statement with the National Institutes of Health, Office of Laboratory Animal Welfare. To ensure compliance, each study protocol was approved by the Institutional Animal Care and Use Committee (IACUC) before the initiation of treatment. Each study was designed to use the fewest number of animals possible consistent with the objective(s) of the study, the scientific needs of the Sponsor, and contemporary scientific standards.

## 3 Results

### 3.1 Characterization of the interaction of narsoplimab with MASP-2

Narsoplimab bound to human MASP-2 with high affinity, with *K*_D_ values of 0.073 nM and 0.062 nM as determined by surface plasmon resonance (**Table 1**) and ELISA (**Table 2**), respectively. The affinity of narsoplimab-related monovalent Fab for human MASP-2 was approximately 100-fold lower: the *K*_D_ was 10.3 nM by surface plasmon resonance (**Table 1**) and 14.9 nM by ELISA (**Table 2**). While narsoplimab and monovalent Fab associated with human MASP-2 at similar rates (k_a_, 3.5 vs 2.6 × 10^6^ M^-1^s^-1^), narsoplimab dissociated approximately 100-fold more slowly than monovalent Fab (k_d_, 2.47 × 10^-2^ vs 2.57 × 10^-4^ s^-1^; **Table 1**), indicating a substantial contribution of avidity to the tight interaction of narsoplimab with MASP-2.

**Table 1:** Kinetics and affinity of narsoplimab or a monovalent Fab binding to MASP-2 as assessed by surface plasmon resonance.

	Surface Plasmon Resonance, mean ± SE			
	N	k_a_ (M^-1^s^-1^)	k_d_ (s^-1^)	*K*_D_ (nM)
Narsoplimab	n=3	3.46 ± 1.1 × 10^6^	2.47 ± 0.55 × 10^-4^	0.073 ± 0.007
Fab	n=4	2.55 ± 0.81 × 10^6^	2.57 ± 0.36 × 10^-2^	10.3 ± 2.8

Fab, antigen-binding fragment; k_a_, association rate constant; k_d_, dissociation rate constant; *K_D_*, dissociation equilibrium constant; MASP-2, mannan-binding lectin-associated serine protease-2.

**Table 2:** Affinity of narsoplimab or a monovalent Fab for MASP-2 as assessed by ELISA.

	ELISA, mean ± SD	
	n	*K*_D_ (nM)
Narsoplimab	n=12	0.062 ± 0.023
Fab	n=2	14.9

ELISA, enzyme-linked immunosorbent assay; Fab, antigen-binding fragment; K_D_, dissociation equilibrium constant; MASP-2, mannan-binding lectin-associated serine protease-2.

**Figure 2:**
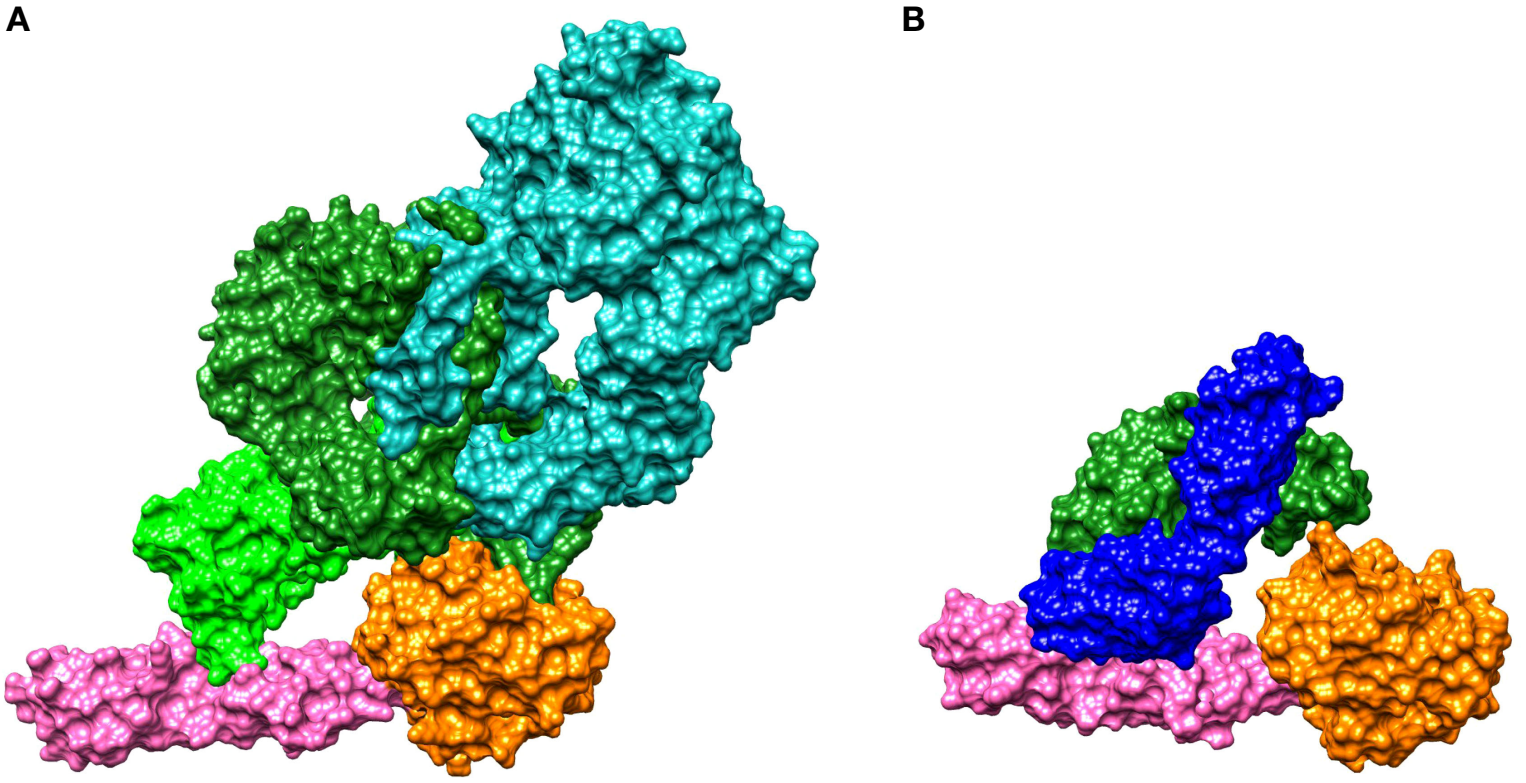
Crystal structures for binding of: **(A)** C4 to MASP-2 and **(B)** the narsoplimab Fab region to MASP-2. MASP-2, which is the key effector enzyme of the lectin pathway, cleaves C4 and C2, activating the complement system (see **Figure 1**). **(A)** The C4 gamma chain (light green) interacts with the CCP1 and CCP2 domains of MASP-2 (magenta), while the C4 alpha chain (dark green) interacts with the MASP-2 serine protease domain (orange). The C4 beta chain is shown in teal. C4 is activated by proteolytic cleavage of the C4 alpha chain, forming C4a and C4b (35). **(B)** Narsoplimab Fab (light chain in blue, heavy chain in green) binds to MASP-2 on the CCP1 and CCP2 domains (magenta) at a distance from the MASP-2 serine protease domain (orange). Thus, the most plausible mechanism for inhibition of MASP-2–dependent complement activation by narsoplimab appears to be direct competition for a C4 substrate binding site on MASP-2 (an exosite), inhibiting the interaction of MASP-2 with C4 substrate and preventing proteolytic activation of C4. Cleavage of C4 otherwise precedes the activation of downstream components of the complement system. CCP, complement control protein; Fab, antigen-binding fragment; MASP, mannan-binding lectin-associated serine protease.

To shed light on the mechanism of MASP-2 inhibition by narsoplimab, we analyzed co-crystal structures for narsoplimab Fab bound to MASP-2 and C4 bound to MASP-2 (Protein Data Bank code 5JPM) (**Figure 2**). From the co-crystal structures, it is evident that narsoplimab and C4 bind to a common site bridging the complement control protein (CCP) domains CCP1 and CCP2 of MASP-2. Hence, narsoplimab appears to inhibit MASP-2 activity by competing with C4 substrate for MASP-2 binding. Interestingly, the active proteolytic site of MASP-2 that cleaves and activates C4 is not directly impacted by narsoplimab binding.

### 3.2 Specificity and selectivity of narsoplimab

The specificity of narsoplimab for human MASP-2 relative to four other serine proteases of the complement system (C1r, C1s, MASP-1, and MASP-3) was assessed by solid-phase ELISA. Narsoplimab bound with high-affinity to zymogen and enzymatically active forms of human MASP-2 (*K*_D_ = 0.062 nM and 0.089 nM, respectively). By contrast, the interaction with C1s exhibited >5000-fold lower affinity (*K*_D_ ~500 nM); no discernable binding to C1r, MASP-1, or MASP-3 was observed at concentrations up to 500 nM (**Table 3**).

**Table 3:** Affinity of narsoplimab for MASP-1, MASP-2, MASP-3, C1r, and C1s.

Antigen	Narsoplimab *K*_D_ (nM)
MASP-1	> 500^a^
Zymogen MASP-2	0.062 ± 0.023^b^
Active MASP-2	0.089 ± 0.012^b^
MASP-3	> 500^a^
C1r	> 500^a^
C1s	~ 500^c^

C, complement component; *K_D_*, dissociation equilibrium constant; MASP, mannan-binding lectin-associated serine protease.

a Narsoplimab at 500 nM yielded less than half of the MASP-2 binding response.

b Mean ± SD; n = 12.

c Narsoplimab at 500 nM yielded approximately half of the MASP-2 binding response.

**Figure 3:**
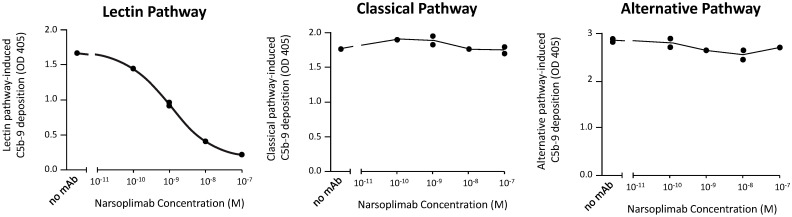
Narsoplimab inhibition of C5b-9 deposition in human serum by the lectin, alternative, or classical pathway. Duplicate narsoplimab samples were pre-incubated with human serum followed by the evaluation of C5b-9 activation under pathway-specific assay conditions, as provided by the Wieslab^®^ total complement screening kit. OD, optical density; mAb, monoclonal antibody.

To assess selectivity, the effects of narsoplimab on lectin, classical, and alternative pathway-dependent activation of C5b-9 were compared using the Wieslab^®^ total complement screening kit. These tests showed that narsoplimab inhibited lectin pathway-dependent activation of C5b-9 in human serum with an IC_50_ of ~1 nM whereas narsoplimab concentrations up to 500 nM had no observable effect on classical pathway-dependent or alternative pathway-dependent activation of C5b-9 in human serum (**Figure 3**). These data demonstrate that narsoplimab binds to MASP-2 with high specificity and selectively inhibits the lectin pathway without affecting the classical pathway or alternative pathway of the complement system.

### 3.3 Pharmacologic activity of narsoplimab *in vitro*

ELISA-based and flow cytometry-based test methods were developed to assess lectin pathway-dependent activation of C3 or C4 under near-physiologic conditions and to characterize the pharmacologic activity of narsoplimab in mice, rabbits, cynomolgus monkeys, and humans. To conduct these evaluations at near-physiologic (90%) serum concentrations, it was necessary to reduce the reaction temperature to 4°C in order to achieve reaction kinetics compatible with standard benchtop plate-based assay procedures. Under these conditions, reaction kinetics were characterized by an initial lag phase followed by a near-linear time-course over 30 to 60 minutes (data not shown). For each species, a reaction time during the linear phase of the reaction that provided a robust lectin pathway activation signal in the absence of narsoplimab, with minimal background complement activation in the presence of excess narsoplimab, was selected for further pharmacologic assessments. Background complement activation was presumably due to classical or alternative pathway activity, particularly for C3 activation.

**Figure 4:**
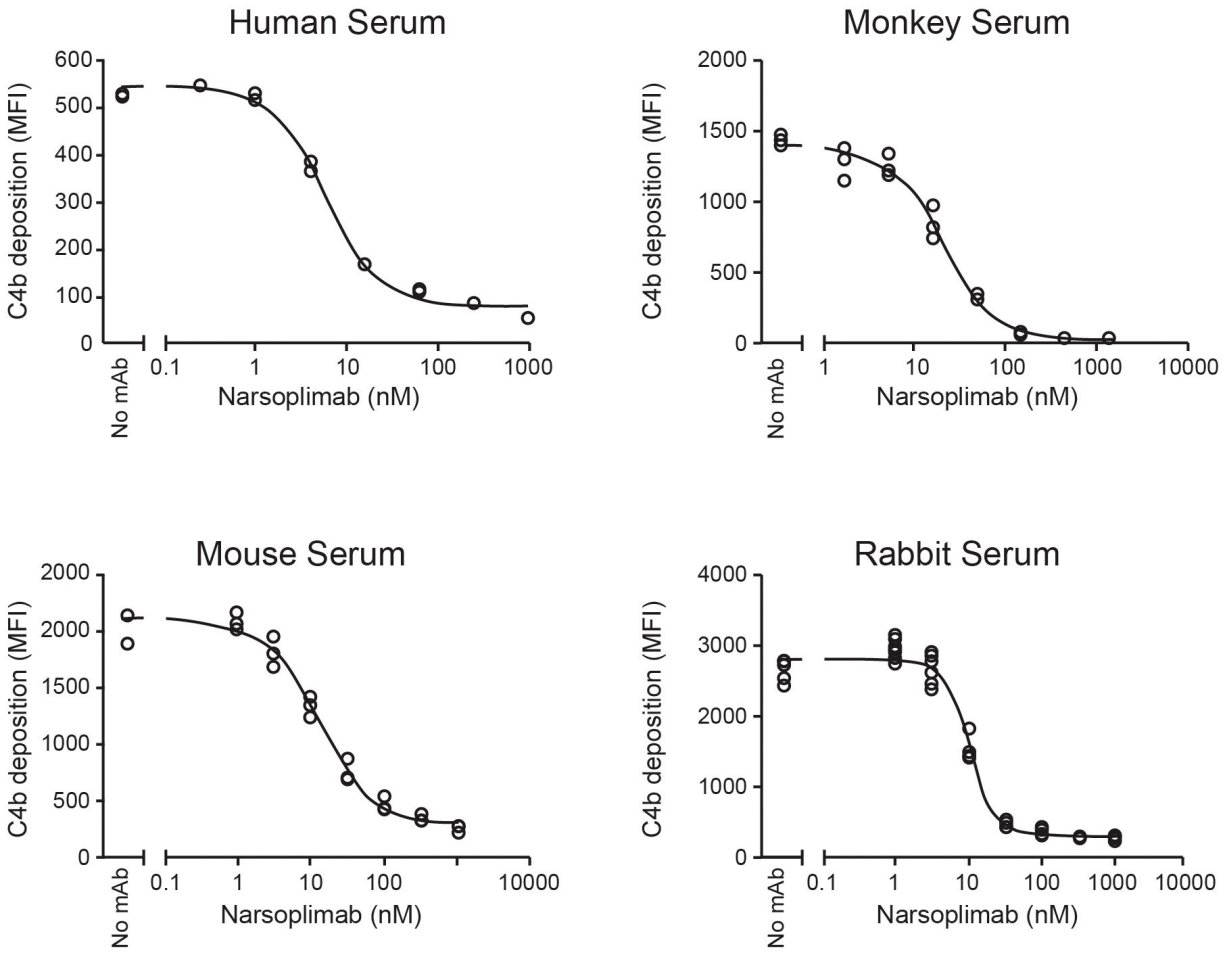
Functional inhibition of lectin-induced C4 activation in 90% serum. mAb, monoclonal antibody; MFI, mean fluorescent intensity.

Optimized conditions for ELISA-based and flow cytometry-based lectin pathway functional assays were applied to *in vitro* pharmacologic characterization of narsoplimab in serum from mice, rabbits, cynomolgus monkeys and humans. Representative examples of inhibition of lectin pathway functional activity assessed by flow cytometry are shown in **Figure 4**, and the data from all experiments are summarized in **Table 4**. Using the flow cytometry assay, 50% inhibition of C4 activation in human, mouse, rabbit, and cynomolgus monkey sera was achieved at 3.4 ± 2.3, 13 ± 1.3, 9.2 ± 2.6, and 33 ± 13 nM, respectively. Where tested, the IC_50_ value for inhibition of C3 activation and inhibition of C4 activation were comparable (**Table 4**). ELISA assays yielded IC_50_ values comparable to the flow cytometry assays, although ELISA assays tended to have greater variability due to the lower signal-to-noise ratios compared with flow cytometry assays (**Supplementary Table 1**). In rat serum, narsoplimab concentrations up to 500 nM achieved <50% inhibition of lectin pathway activation using the ELISA assay, indicating that narsoplimab does not have appreciable pharmacologic activity in rats (data not shown).

**Table 4:** IC_50_ values for lectin pathway inhibition in mice, rabbits, cynomolgus monkeys, and humans obtained using the flow cytometry assay in comparison to the ELISA assay.

Species	Flow cytometry assay (mean ± SD; nM)		ELISA assay (mean ± SD; nM)	
	IC_50_ C4 activation	IC_50_ C3 activation	IC_50_ C4 activation	IC_50_ C3 activation
Mice	13 ± 1.3	15 ± 2.5	18 ± 1.5	18 ± 2.3
Rabbits	9.2 ± 2.6	ND	21	ND
Monkeys	33 ± 13	ND	75 ± 30	123 ± 19
Humans	3.4 ± 2.3	4.3 ± 2.3	2.4 ± 1.6	2.6 ± 1.2

ND, not determined; C, complement component; ELISA, enzyme-linked immunosorbent assay; IC_50_, half maximal inhibitory concentration; SD, standard deviation.

### 3.4 Pharmacokinetic/pharmacodynamic relationship of narsoplimab in cynomolgus monkeys

**Figure 5:**
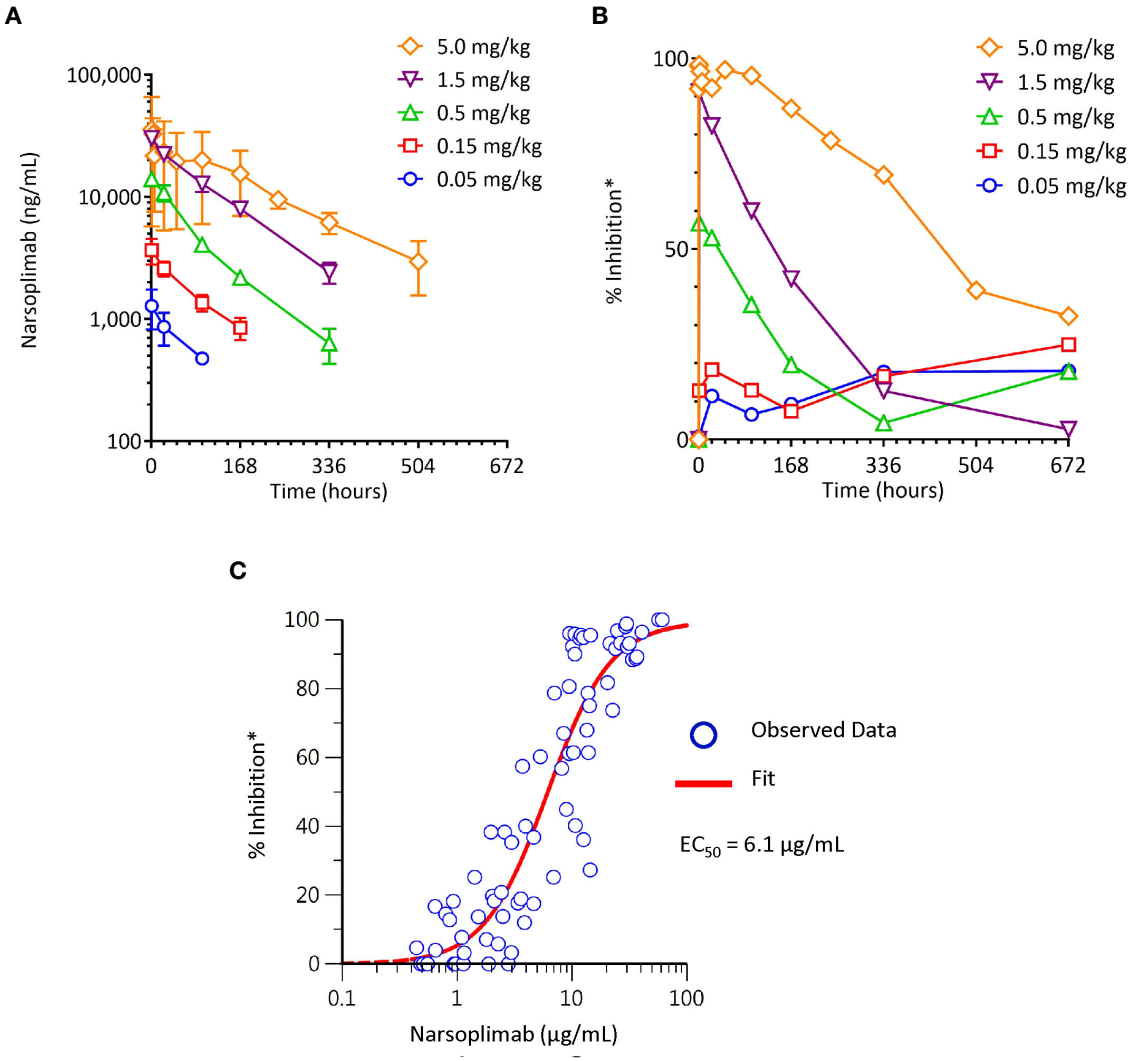
Pharmacokinetics and pharmacodynamics of narsoplimab in cynomolgus monkeys after a single IV infusion of narsoplimab. **(A)** Dose-dependent exposure after a single IV dose. **(B)** Dose-related pharmacodynamic effect. **(C)** Concentration-dependent pharmacodynamic effect. * % inhibition of the lectin pathway was determined ex vivo by mannan-induced C4 activation with an ELISA assay. EC_50_, half-maximal effective concentration; IV, intravenous.

To characterize the pharmacodynamic response to narsoplimab in primates, the time-course and dose-response of lectin pathway inhibition measured *ex vivo* was evaluated in cynomolgus monkeys treated with varying doses of narsoplimab. Narsoplimab concentrations were also determined to define the PK profile and to characterize the PK/PD relationship in cynomolgus monkeys. Narsoplimab exposure increased over the dose range evaluated (**Figure 5A**), with approximately dose-proportional increases in exposure from 0.05 to 1.5 mg/kg and a less-than-proportional increase at 5 mg/kg. The terminal half-life was approximately 3 to 8 days.

As expected, increasing doses were associated with corresponding increases in the magnitude and duration of the pharmacodynamic response (**Figure 5B**). While no appreciable pharmacodynamic response (% inhibition of lectin pathway activity measured *ex vivo* in post-dose serum samples relative to lectin pathway activity measured at baseline) was seen after 0.05 or 0.15 mg/kg administration of IV narsoplimab, clear dose-related pharmacodynamic responses were observed in the dose range of 0.5 to 5 mg/kg IV. Single dose IV administration of 0.5 or 1.5 mg/kg of narsoplimab to cynomolgus monkeys resulted in approximately 60% and 90% inhibition, respectively, of *ex vivo* lectin pathway activity immediately after administration, followed by a gradual decline of inhibition over time. A single dose administration of 5 mg/kg of narsoplimab IV in cynomolgus monkeys resulted in nearly complete inhibition of lectin pathway activity that lasted for more than 1 week followed by a slow and gradual decline of inhibition over time.

Further analysis of pharmacokinetic and pharmacodynamic data revealed a well-defined relationship between narsoplimab serum concentration and pharmacodynamic response, with an *ex vivo* EC_50_ value of ~6 µg/mL (**Figure 5C**). This *ex vivo* EC_50_ value in cynomolgus monkeys was in good agreement with the *in vitro* IC_50_ value for inhibition of lectin pathway activity in the same species (33 ± 13 nM, ~5 µg/mL), indicating that *in vitro* functional potency data predict *in vivo* target engagement as a function of narsoplimab serum concentrations in primates. Based on the substantially greater functional potency of narsoplimab in humans relative to monkeys (IC_50_ of 3.4 nM [~0.5 µg/mL] for humans compared to 33 nM [~5 µg/mL] for cynomolgus monkeys), it is expected that pharmacodynamic responses can be achieved in humans at approximately 10-fold lower drug concentrations and a correspondingly lower dose.

## 4 Discussion

We developed narsoplimab, a fully human IgG4 monoclonal antibody, to bind to and specifically inhibit MASP-2, the effector enzyme of the lectin pathway (21). Lectin pathway-induced C3 and C5 activation products are responsible for platelet and endothelial cell activation, leukocyte recruitment, and tissue injury (6). MASP-2 also activates the coagulation cascade through cleavage of prothrombin to thrombin (48, 49) and activation of Factor XII (50). Deficiency or blockade of MASP-2 in experimental models has been shown to have beneficial effects in ischemic reperfusion injury (21, 37–39), transplantation (39), rheumatoid arthritis (51), TMA (52), pneumococcal meningitis (53), SARS-Cov-2 infection (54), sickle cell disease (55), and renal fibrosis (40).

In this series of preclinical studies, we analyzed the *in vitro* pharmacologic properties of narsoplimab and its pharmacokinetic and pharmacodynamic properties in primates. *In vitro* binding studies demonstrated that intact narsoplimab interacts with human MASP-2 in high avidity: its affinity for MASP-2 was approximately 100-fold greater than was the affinity of the monovalent Fab. The higher affinity with intact narsoplimab compared to Fab was almost exclusively due to the much slower dissociation of the antibody-antigen complex. These results suggest that once narsoplimab is bound to MASP-2, it forms a high-avidity complex, indicating that functional bivalency substantially contributes to narsoplimab’s interaction with MASP-2. The functional significance of bivalency is further supported by functional activity data, which showed that the monovalent scFv precursor to narsoplimab exhibited approximately 50-fold lower functional potency in lectin pathway assays conducted in dilute serum (IC_50_ for scFv ~30 nM compared to ~0.6 nM for narsoplimab [data not shown]). *In vivo*, such a high-avidity complex may arise from the binding of each Fab domain of a single narsoplimab antibody to the CCP1 and CCP2 domains of two adjacent MASP-2 molecules. To safeguard against potential loss of bivalency, narsoplimab includes the S228P mutation in the hinge region of the heavy chain, which is known to stabilize the inter-chain disulfide bridge and reduces the formation of functionally monovalent IgG4 half antibodies (56).

ELISA studies demonstrated that narsoplimab had more than 5000-fold specificity for binding to both zymogen and enzymatically active forms of human MASP-2 (*K_D_* of 0.062 nM and 0.089 nM, respectively) compared with four closely related serine proteases of the complement system, specifically MASP-1, MASP-3, C1r, and C1s (each with *K_D_* of ≥500 nM). The high affinity and specificity of narsoplimab to bind human MASP-2 resulted in selective inhibition of the lectin pathway based on Wieslab ® assay results that evaluate C5b-9 activation levels under pathway-specific assay conditions in dilute human serum samples. Narsoplimab inhibited C5b-9 activation under lectin pathway-specific assay conditions with IC_50_ of approximately 1 nM while leaving the classical and alternative pathways of complement unaffected at concentrations up to 500 nM.

To further characterize the pharmacologic activity of narsoplimab, we developed and optimized ELISA-based and flow cytometry-based methods to evaluate lectin-dependent C3 and C4 activation at near-physiologic serum concentrations. While both assay methods yielded comparable IC_50_ values for lectin pathway inhibition by narsoplimab, the flow cytometry assay provided greater dynamic range and signal-to-noise ratio, and hence greater data robustness, compared with the ELISA assay. Both methods demonstrated that narsoplimab potently inhibits lectin-dependent C3 and C4 activation with an IC_50_ value of approximately 3 nM in minimally diluted human serum. This IC_50_ value is in the range of the MASP-2 concentration present in human serum (approximately 400 ng/mL or 5 nM), which suggests that the potency of narsoplimab to inhibit the lectin pathway at physiologic target concentrations may be driven, at least in part, by stoichiometry. Narsoplimab also inhibited lectin-dependent activation in mouse, rabbit, and cynomolgus monkey sera, albeit with lower potency. While differences in the MASP-2 protein sequence between mice and rabbits compared to humans (~80% sequence identity between mice and humans) may account for the reduced functional potency of narsoplimab in these non-human species, the 10-fold higher IC_50_ value in cynomolgus monkey compared to humans was surprising given the 95% degree of sequence identity between cynomolgus monkeys and humans. Since the IC_50_ value for MASP-2 inhibition in human serum approximates the human MASP-2 concentration, it is possible that the higher IC_50_ value for MASP-2 inhibition in cynomolgus monkey serum is the result of higher serum MASP-2 levels in cynomolgus monkeys compared to humans.

We developed and optimized *ex vivo* methods to assess changes in serum lectin pathway activity in primates: we collected serial blood samples before and after exposure of the animals to narsoplimab *in vivo*, exposed those samples to mannan, and measured C4 activation *ex vivo*. A clear, dose-related pharmacodynamic response occurred in the dose range of 0.5 to 5 mg/kg in monkeys, as demonstrated by a dose-related increase in extent and duration of the inhibition of lectin pathway activity. A well-defined pharmacokinetic/pharmacodynamic relationship was seen between narsoplimab serum concentrations and pharmacodynamic response, with modeling indicating an EC_50_ value of ~6.1 µg/mL (~40 nM). This *ex vivo* EC_50_ value was comparable to the IC_50_ value for lectin pathway inhibition determined *in vitro* (33 nM; ~5 μg/mL), demonstrating good agreement between *in vitro* and *ex vivo* assessments of narsoplimab’s inhibition of the lectin pathway. Both the pharmacokinetic profile (exposure and terminal half-life) and its pharmacodynamic effect in primates (near-complete inhibition of lectin pathway activity at 5 mg/kg IV persisting for greater than 1 week) support the investigation of once-weekly dosing of narsoplimab in humans.

Given these results in preclinical models, as well as a growing body of literature invoking the lectin pathway in multiple pathophysiological processes, narsoplimab treatment is being investigated in clinical trials of lectin pathway-mediated conditions where MASP-2 is believed to play a key role in pathophysiology. In hematopoietic stem cell transplantation-associated thrombotic microangiopathy (HSCT-TMA), plasma MASP-2 levels are elevated versus healthy controls, and narsoplimab has been shown to reduce HSCT-TMA plasma-mediated endothelial damage in a tissue culture model (52). In a pivotal clinical trial for HSCT-TMA, once-weekly narsoplimab treatment resulted in 61% response rate based on improvement in laboratory TMA markers and clinical benefit, indicating clinically relevant resolution of HSCT-TMA pathophysiology (57). Based on this evidence, narsoplimab appears to be beneficial in the treatment of lectin pathway-mediated diseases.

In conclusion, we developed narsoplimab to specifically bind to MASP-2 and to selectively inhibit activation of the lectin pathway of complement. In this series of preclinical discovery studies, we showed that narsoplimab demonstrates high affinity and selectivity for MASP-2. Once bound, narsoplimab forms a high-avidity complex with MASP-2 due to its functional bivalency. Selective inhibition of MASP-2 by narsoplimab results in functional inhibition of lectin-dependent complement activation, without affecting classical or alternative complement pathways. To characterize the *in vitro* pharmacologic activity of narsoplimab under near-physiologic conditions, we employed newly developed assays evaluating lectin pathway-mediated C4 activation in minimally diluted serum samples and adapted the same methodologies for *ex vivo* pharmacodynamic response assessments. Using these methods, we showed in primates that narsoplimab demonstrates dose-dependent and concentration-related inhibition of the lectin pathway. Based on these findings, clinical studies have evaluated the effects of narsoplimab on MASP-2–dependent complement activation in human subjects, including patients with HSCT-TMA.

## Data availability statement

The original contributions presented in the study are included in the article/**Supplementary Material**. Further inquiries can be directed to the corresponding author.

## Ethics statement

Ethical approval was not required for the studies on humans in accordance with the local legislation and institutional requirements because only commercially available established cell lines were used. The animal study was approved by MPI Research, Inc. 54943 North Main St., Mattawan, MI 49071. The study was conducted in accordance with the local legislation and institutional requirements.

## Author contributions

TD: Conceptualization, Formal Analysis, Investigation, Methodology, Project administration, Supervision, Writing – original draft, Writing – review & editing, Data curation. SY: Formal Analysis, Investigation, Writing – review & editing, Methodology. WC: Formal Analysis, Investigation, Writing – review & editing, Conceptualization.
