# Supplementary material for: Development and characterization of narsoplimab, a selective MASP-2 inhibitor, for the treatment of lectin-pathway–mediated disorders
Source: Front Immunol. 2023 Nov 8;14:1297352. doi: 10.3389/fimmu.2023.1297352 (PMC10663225; doi:10.3389/fimmu.2023.1297352)
Supplement: Supplementary file 1 [file Table_1.pdf]

Supplementary Materials

Supplementary Table 1. Narsoplimab signal-to-noise ratios (mean ± SD) for lectin pathway inhibition by flow cytometry and ELISA assays

	ELISA assay (mean ± SD)		Flow cytometry assay (mean ± SD)	
**Species**	**Assay ratio[a] C4 activation**	**Assay ratio[a] C3 activation**	**Assay ratio[a] C4 activation**	**Assay ratio[a] C3 activation**
Mice	7.3 ± 1.3	4.6 ± 0.9	9.2 ± 2.7	5.3 ± 0.9
Rabbits	2.8 to 3.4	-	7.8 ± 1.2	-
Monkeys	2.9 ± 1.8	4.1 ± 0.9	71 ± 28	-

- = not determined; C = complement component; ELISA = enzyme-linked immunosorbent assay; IC_{50} = half maximal inhibitory concentration; SD = standard deviation.

[a] Assay ratios were calculated as top/bottom of the data fit for IC_{50} obtained using the 4-parameter logistic regression.
